# Carotid near-occlusion diagnostics and its consequences: A systematic review

**DOI:** 10.1093/esj/23969873251355158

**Published:** 2026-01-01

**Authors:** Elias Johansson, Intisaar Barud, Sofia Strömberg

**Affiliations:** Neuroscience and Physiology, Department of Clinical Neuroscience, Gothenburg University, Gothenburg, Sweden; Neuroscience and Physiology, Department of Clinical Neuroscience, Gothenburg University, Gothenburg, Sweden; Institute of Medicine, Department of Molecular and Clinical Medicine, Gothenburg University, Gothenburg, Sweden

**Keywords:** Stroke, carotid stenosis, near-occlusion, ultrasound, CT-angiography, phase-contrast MRI

## Abstract

**Introduction:**

To summarize carotid near-occlusion (CNO) diagnostics and its consequences on epidemiology and management.

**Materials and methods:**

A systematic search of PubMed using 19 known synonyms for CNO was performed. Diagnostic analyses of CNO were assessed. Epidemiological and management analyses were based on how the CNO diagnostics was conducted, with diagnostics resembling large trials considered “good.”

**Results:**

CNO can be diagnosed with several modalities and approaches (interpretation or measurements). Interpretation of angiography is the reference standard but is not feasible for routine use. Of feasible methods, flow measurements with phase-contrast magnetic resonance imaging (PC-MRI) were considerably better than other alternatives when assessed blinded: 90%–100% sensitive and 99%–100% specific and inter-rater kappa 0.98–1.0. CNO was consistently common (30% of ⩾50% stenosis) in studies with “good” CNO diagnostics but was also often described as rare. Symptomatic CNO have no benefit with revascularization in studies with “good” CNO diagnostics, which foremost applies to the moderate subtype (without full collapse). The more severe CNO subtype (with full collapse) seems to have a very high risk of stroke within the first 2 days, but revascularization performed sufficiently early to prevent this has never been assessed.

**Discussion:**

CNO diagnostics is difficult and that CNO is perceived as rare by many is likely due to poor diagnostics. Such poor diagnostics also likely result in unnecessary surgeries for many symptomatic CNOs.

**Conclusion:**

CNO is a common variant of carotid stenosis. New diagnostic methods (especially PC-MRI) should be introduced, possibly after validation of its prognostic impact in a randomized trial.

## Graphical Abstract

**Graphical abstract:**
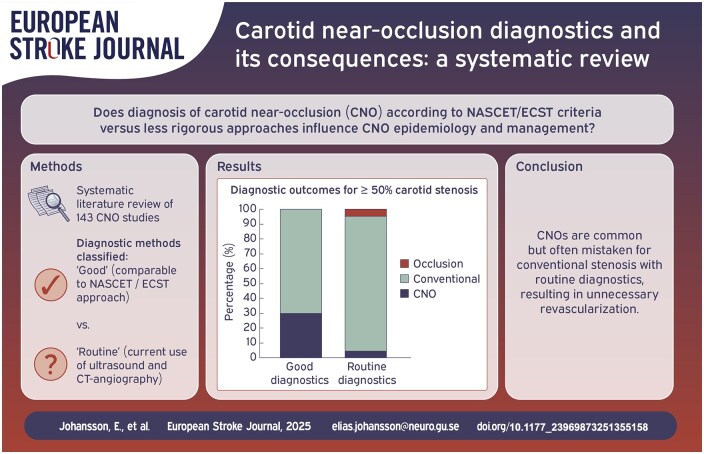

## Introduction

**Figure 1.**
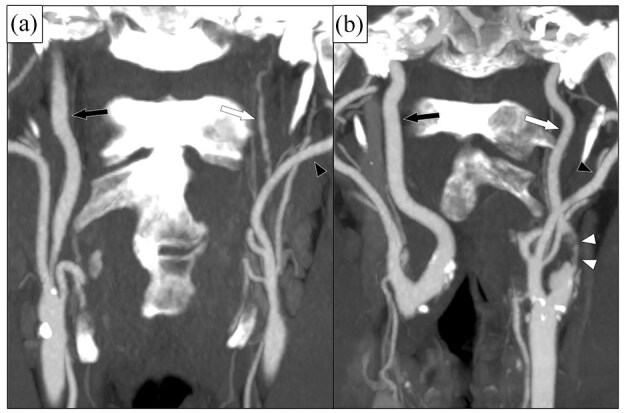
Two cases of left-sided CNO, coronal view on CTA. (a) CNO with full collapse. After a severe stenosis (not in plane), the distal ICA (white arrow) is very small (threadlike), clearly smaller than contralateral ICA (black arrow) and ipsilateral ECA (black arrowhead). (b) CNO without full collapse. After a severe and quite long stenosis (white arrowheads), the distal ICA (white arrow) is normal-appearing, but smaller than contralateral ICA (black arrow) and similar as ipsilateral ECA (black arrowhead).

Carotid near-occlusion (CNO) is a severe atherosclerotic stenosis of the proximal internal carotid artery (ICA) causing a distal ICA diameter reduction (“collapse”),^1–3^ whereas conventional stenoses are those not causing distal ICA narrowing or collapse. Guidelines recommend a foremost conservative treatment approach for symptomatic CNO.^4–7^ This recommendation is based on major trials where the participants with CNO rarely (6%) had the severe variant of CNO (CNO with full collapse, Figure 1(a)).^1^ Instead, almost all participants with CNO (94%) had the moderate variant of CNO, that is, CNO without full collapse (Figure 1(b)).^1^ When grading carotid stenosis, it is crucial to first assess if the case have CNO and only grade conventional stenoses with percent.^1–3^

As expressed in the only review of CNO diagnostics presented in 2016,^2,3^ many studies, including relevant diagnostic studies, do not include CNO at all or have limited the definition of CNO to the severe variant. Hence, diagnostics that only work for a narrow CNO definitions are still use, that is, are not applicable to trial definitions or guideline recommendations. How CNO definition affects epidemiological assessments has never been summarized. Furthermore, since this 2016 review, there has been significant developments for CNO diagnostics and management. The diagnostics have not been summarized and while the management studies have been summarized in several reviews and meta-analyses,^8–12^ these did not assess the underlying CNO definition nor separated finding for CNO with and without full collapse. We therefore undertook systematic review with a two-part aim: Summarize the CNO diagnostic field and, more importantly, describe the impact of varying CNO definitions and diagnostics on the epidemiology and management of CNO.

## Method

**Figure 2.**
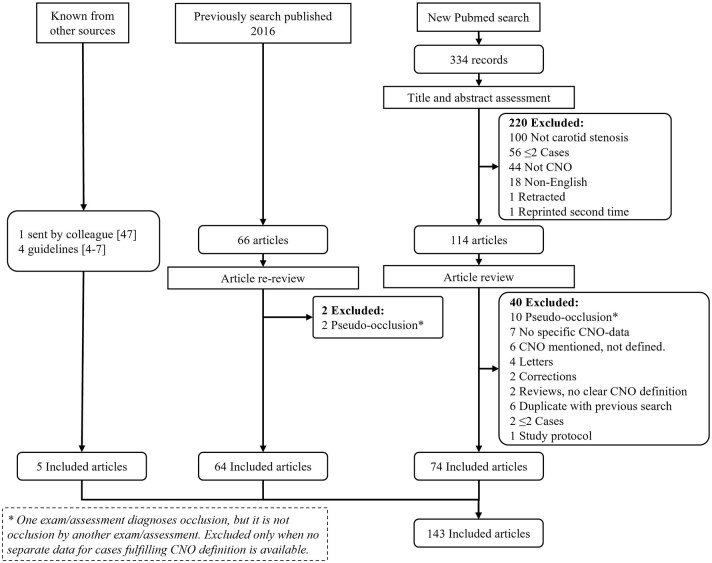
Flow chart. The new Pubmed search was conducted June 5, 2024 for “carotid near-occlusion,” “carotid pseudo-occlusion,” “carotid string sign,” “carotid slim sign,” “carotid critical stenosis,” “small distal carotid artery,” “narrow distal carotid artery,” “carotid preocclusive stenosis,” “carotid pre occlusive stenosis,” “carotid subtotal stenosis,” “carotid sub total stenosis,” “carotid subtotal occlusion,” “carotid sub total occlusion,” “carotid functional occlusion,” “carotid sub-occlusion,” “carotid hypoplasia,” “carotid incomplete occlusion,” and “carotid hairline” between 2015 to date of search. Also, “Carotid near-total occlusion,” was searched without time restriction (as it was not included in previous search^2^).

We followed PRISMA guidelines for reporting systematic reviews. We used references from a previous systematic search of Pubmed, initially presented in 2016.^2,3^ This Pubmed search was repeated for the period January 2015–June 2024 and an added term “carotid near-total occlusion”^13^ was searched (all 19 CNO synonyms we have encountered) without time restriction (Figure 2). The search was assessed by two observers. Inclusion criteria were data for >2 CNO cases or reviews/editorials/guidelines of CNO, and English language. For inclusion in main diagnostic and management assessments, at least 10 CNO cases were required. For study selection, any use of a CNO term/synonym was accepted except if an atherosclerotic stenosis caused a small distal artery was intended. In the current search, this was foremost applicable to “pseudo-occlusion” which has been a term that has seemingly shifted meaning from CNO (several such articles in the 2016 review^2^) to “erroneous occlusion diagnosis.”

### Definitions

One reviewer (EJ) with extensive CNO diagnostic experience assessed each study for whether the CNO definition reasonably adhered to the CNO definition of the pooled analysis of North American Symptomatic Carotid Endarterectomy trial (NASCET) and European Carotid Surgery Trial (ECST).^1^ That is, a severe atherosclerotic stenosis in the common carotid artery or proximal extracranial ICA that causes the distal extracranial ICA to reduce in diameter (collapse). The collapse has to be visible but does not need to be extensive; extensive collapse (full collapse) is rather a subtype of CNO. These three CNO diagnostics criteria were used: (1) “Good,” which reasonably adheres to the NASCET/ECST trial definitions. Both criteria 1A and 1B were required: (1A) a clear indication that all CNOs (both with and without full collapse) were intended in any part of the manuscript, and (1B) no arbitrary additions to previously established criteria. (2) “Restrictive,” which likely mistake some CNOs for conventional stenosis. This is similar to “good” diagnostics but failing either 1A or 1B. (3) “Full collapse alone,” where there is no indication that CNO without full collapse is studied. Either criteria 3A, 3B, or 3C were required: (3A) Actively presenting that only full collapse was assessed by excluding identified CNO without full collapse, (3B) presenting angiographic criteria that reasonably intends to only assess full collapse (such as “string sign”), or (3C) using other diagnostic criteria that are known to only or almost only include CNO with full collapse (defined by review findings, presented below). Also, one relevant study^14^ used diagnostics that obviously led to that some conventional stenosis being categorized as CNO, this is presented separately in context. The pooled analysis from which this definition is derived is henceforth referred to as “NASCET/ECST.” All stenosis grading aspects in this review refer to NASCET criteria.^15^

### Statistics

Prevalence estimates were considered consistent within a group of studies when all studies were within ±10 absolute percent of the mean. Where relevant, we calculated 95% confidence intervals based on Poission distribution and two-sided χ^2^-test from presented data with IBM SPSS 28.0.

## Findings

We identified 143 studies about CNO. The diagnostics used since 2005 (after NASCET/ECST) is summarized in the Supplemental Material.

### Studies of CNO diagnostics

Of 44 diagnostic studies, 11 assessed the separation of CNO (without full collapse) and conventional stenosis, summarized in Table 1 and described in detail in Supplemental Table 1. Remaining 33 studies are summarized in Supplemental Table 2. Separation of CNO from conventional stenosis can be with three different diagnostic approaches, detailed in Supplemental Table 3. Of diagnostic modalities using measurement criteria, phase-contrast magnetic resonance imaging (PC-MRI) stands out for great accuracy (90%–100% sensitive, 99%–100% specific), even when assessed blinded and with excellent interrater reliability (kappa 0.98–1.0).^16,17^ CTA measurements have similar diagnostic performance as PC-MRI when assessed non-blinded (90%–100% sensitive and 96%–100% specific), but not when assessed blinded (79% sensitive and 89% specific).^18–20^ Various approaches have been used for US, but all have poor diagnostic performance (Table 1), detailed in the Supplemental Material.

**Table 1.** Diagnostic studies for separating all CNO and conventional stenosis.

Article	Modality	Blinded	*n*	Approach	Sensitivity	Specificity
Using “good” diagnostics on the reference test						
Fox 2005^1^	CA	Yes	32	IC	91	94
Rothwell 2000^21^	CA	No	3017	MC	82	99.8
Bartlett 2006^18^	CTA	No	240	MC	92	96
Johansson 2020^19^	CTA	No	358	MC	93	97
Manrique-Zegarra 2022^20^	CTA	Yes	51	MC	79^b^	89^b^
Manrique-Zegarra 2022^20^	CTA	Yes	51	IC	77^b^	83^b^
Johansson 2021^16^	PC-MRI	Yes	29	MC	100	100
Holmgren 2024^17^	PC-MRI	Yes	239	MC	84	98
Holmgren 2024^17^	PC-MRI	Yes	239	MC	90	99
Khangure 2018^22^	US	Yes	136	MC	13	100
Khangure 2018^22^	US	Yes	118	MC	98	53
Johansson 2019^23^	US	Yes	60	MC	63	94
Palacios-Mendoza 2020^24^	US	Yes	135	IC	44	NA
Johansson 2021^25^	US	Yes	445	MC	22	99.3
Johansson 2021^25^	US	Yes	337	MC	75	75
Using “restrictive” diagnostics on the reference test: No studies						
Using “Only full collapse” diagnostics on the reference test: Not applicable^a^						

CA: conventional angiography; CCA: common carotid artery; CNO: carotid near-occlusion; CTA: computed tomography angiography; EDV: end-diastolic velocity; IC: interpretive criteria; ICA: internal carotid artery; MC: measurement criteria; NA: not available; PC-MRI: phase-contrast MRI; PI: pulsatility index; PSV: peak systolic velocity; US: ultrasound.

For further details, please see Supplemental Table 1.

^a^Per definition, studies assessing FC alone do not asses all CNOs.

^b^Values presented for two observers separately, this is the mean of those two values.

CNO is separated from occlusion by establishing patency. Many included studies assessed methods that were novel at the time (especially US methods), but standard today (Supplemental Table 2). Approximately 15% of CNOs can be misidentified as occlusions using ultrasound, CTA, and PC-MRI due to factors that vary between these modalities, which are detailed in the supplement.

For approaches with low sensitivity and high specificity, 79% (136/173) of false negative CNOs were graded as conventional stenosis (remaining as occlusions) when CTAs were assessed in routine practice.^19^ For traditional low flow approach to US, this was 84% (107/127).^22,25^ In more sensitive US methods, but with lower or unknown specificity, 45% (41/91) of false negative CNOs were graded as conventional stenosis.^23,24^ How these results were used to create the graphical abstract is detailed in the Supplemental Material.

### Consequences of CNO diagnostics on CNO epidemiology

Prevalence of CNO was reported in 35 non-duplicate studies (Table 2, Supplemental Tables 4–6). The CNO prevalence varied widely between studies, seemingly mostly due to CNO definition and choice of denominator, and to some extent study type (patient series or revascularization series). Many studies mixed symptomatic and asymptomatic cases, but this seemed to have a small impact on prevalence. Studies that assessed patient series with “good” diagnostics were consistent and found a mean CNO prevalence of 30% among symptomatic ⩾50% stenosis (50% among ⩾70% stenosis). CEA/CAS series with “good” diagnostics had overall a slightly lower prevalence and were less consistent.

**Table 2.** Summary of epidemiological studies based on CNO diagnostics.

Diagnostics	Denominator	Prevalence of CNO % (n/N)	*n* of studies	Consistent between studies^a^
Patient series (not selected by CEA/CAS)				
Good	⩾50% stenosis	30 (316/1059)	5	Yes
	⩾70% stenosis	50 (190/381)	4	Yes
Restrictive^b^	⩾70% stenosis	10 (34/337)	1	-
Only full collapse	⩾50% stenosis	5 (68/1270)	6	Almost^c^
	⩾70% stenosis	13 (14/108)	2	Almost^c^
CEA/CAS series				
Good	⩾50% stenosis	13 (388/3077)	2	No
	⩾70% stenosis	26 (483/1839)	5	No
	Performed CEA/CAS	22 (369/1685)	6	No
Restrictive^b^	⩾70% stenosis	17 (130/777)	3	No
	Performed CEA/CAS	11 (247/2186)	3	No
Only full collapse	Performed CEA/CAS	3 (116/3369)	5	Yes

^a^All studies within ±10 absolute percent of mean.

^b^Uses variation of criteria that likely resulted in that some CNOs without full collapse were categorized as conventional stenosis (i.e. neither full collapse alone nor likely that conventional stenoses were mistaken for CNOs), detailed in Supplemental Table 4.

^c^All studies with “only full collapse” had ⩽7% prevalence with one exception.^26^ The exception only assessed patients with stroke, and stroke is known to be more common in CNO with full collapse.^27^

### Consequences of CNO diagnostics on CNO management studies

#### Prognosis with best medical therapy (BMT) without or before CEA/CAS

Seven studies have assessed prognosis with BMT in symptomatic CNO, of which five used “good” diagnostics (Table 3, Supplemental Table 7). In long-term studies, no benefit of CEA/CAS was seen (*p* ⩾ 0.82) and full collapse status do not impact these findings.^1,28^ In short-term studies, there seemed to be a high risk of stroke recurrence within 2 days of presenting event in CNO with full collapse, but with mixed results (Table 3). An individual patient data meta-analysis is ongoing for these three studies. Conversely, the three studies without “good” CNO diagnostics all seemed to suggest benefit with CEA/CAS. Their additional design issues are presented in Supplemental Table 7.

**Table 3.** Summary of management studies for symptomatic CNO.

Study	Diagnostics	Design	*n*	FC	Outcome	Group 1	Group 2	*p*
Studies with “good” diagnostics, comparing with BMT, all without obvious design issues								
NASCET /ECST^1,15^	Good	Randomized	148 + 114	6%	IIS + PO, 5 years	CEA + BMT: 17%	BMT: 15%	0.90
García-Pastor et al.^28^	Good	Observational	70 + 71	36%	IIS + PO, 2 years	CEA/CAS + BMT: 10%	BMT: 12%	0.82
Studies with “good” diagnostics, comparing CNO with and without FC, all without obvious design issues								
García-Pastor et al.^29^	Good	Observational	41 + 73	36%	IIS, 90 days^a^	FC: 0%	Not FC: 6%	0.15
Johansson et al.^30^	Good	Observational	46 + 70	40%	IIS, 2 + 28 days^a^	FC: 22% + 36%	Not FC: 3% + 3%	<0.001
Henze et al.^31^	Good	Observational	26 + 92	22%	IIS, 2 + 28 days^a^	FC: 16% + 22%	Not FC: 3% + 16%	0.22^b^
Studies without “good” diagnostics and also had obvious design issues								
O’Leary^32^	FC alone	Observational	23 + 9	100%	IIS + PO, 3 years	CEA + BMT: 9%	BMT: 33%	NA
Song et al.^33^	Restrictive	Observational	48 + 31	NA	IIS or TIA + PO, 1 year	CAS + BMT: 2%	BMT: 19%	0.026
Radak et al.^14^	Too inclusive^c^	Observational	259 + 50	NA	IIS + PO, 1 year	CEA + BMT: 2%	BMT: 14%	<0.001

BMT: best medical treatment; CAOS: CAsi Oclusión Síntomatica; CAS: carotid artery stenting; CEA: carotid endarterectomy; CNO: carotid near-occlusion; CS: conventional stenosis; ECST: European Carotid Stenosis Trial; FC: full collapse; IIS: ipsilateral ischemic stroke; NASCET: North American symptomatic carotid endarterectomy trial; PO: perioperative risk (30-day risk of stroke or death).

^a^Counted from after presenting event until CEA/CAS or until end of follow-up for those not yet treated.

^b^2-days presented separately: *p* = 0.01.

^c^Clearly includes cases with CS in CNO group, detailed in Supplemental Table 7.

Prognosis for asymptomatic CNO has only been presented for 22 patients, none had a stroke during 5-year follow-up.^34^

#### Perioperative risk and long-term risk of stroke after CEA/CAS

We found 30 studies assessing 30-day risk of stroke or death after CEA/CAS with >10 cases, of which 18 also presented data on long-term risk of ipsilateral ischemic stroke after CEA/CAS (Supplemental Tables 8 and 9, Supplemental Figures 1–4). The overall perioperative risk for was 4.2% (65/1552) and annual risk of stroke after CEA/CAS 1.0% (21/2130). CNO with full collapse seemed to have a somewhat increased 30-day risk of stroke or death with CEA (6.4%, 21/326) with CEA, not seen for CAS (2.1%, 4/193, Supplemental Table 9). Beyond this, the CNO diagnostics did not seem to affect these risk assessments. Prevalence of hyperperfusion syndrome varied considerably with used CNO diagnostics but could also be well explained by very varying definitions (detailed in Supplemental Material).

## Discussion

The main findings in this review are that different diagnostics approaches for CNO have markedly different accuracy and how CNO is diagnosed has marked effect on epidemiological and management study findings. While it is not novel that CNO has diagnostic issues, 80% of the diagnostic analyses in this review were not available in 2016 (in the last similar review)^2,3^ and the approach to assess studies separately based on their CNO diagnostics is a novel approach. The main implications are an understanding that we might not have diagnosed CNOs well in the past, leading to misapplication of management study findings – and this should be improved.

### CNO diagnostics and definitions

The diagnostics of CNO is the practical application of a CNO definition. One of the main strengths with this review is the assessment of the underlying diagnostics in each study, leading to nuanced findings. Otherwise, CNO is easily misunderstood, ignored or mistaken to only include full collapse, making the field difficult to understand, which is further presented in the Supplemental Material.

### CNO diagnostics: What works and what is used

The reference method for separating CNO and conventional stenosis is feature interpretation of angiography, as this was used in NASCET/ECST.^1^ It is also the reference test in most diagnostic studies (Supplemental Table 1). How angiographic exams are interpreted seems to be of much greater importance than modality (CTA vs CA, magnetic resonance imaging less studied): In Johansson et al. 65% (68/104) of CNOs were mistaken for conventional stenosis in the routine assessment compared to expert-assessment of the same scan (detailed in Supplemental Material).^19^ While CA is the most frequently used modality in recent CNO studies, it is rarely assessed with feature interpretation, see Supplemental Material for details.

While feature interpretation can be used in studies where experts assess many cases during a short time-span, it is likely difficult to use in routine diagnostics as cases are usually spread out over many assessors and over time, especially in smaller hospitals. While a formal assessment of this is lacking, the best assessment we can do at this stage is that feature interpretation is not a good solution for the unmet need of good CNO diagnostics in routine practice, which is further discussed in the Supplemental Material. Hence, there is a need for easy-to-use yet accurate methods for routine use. Here, PC-MRI stands out as the best method to diagnose CNO given accuracy (shown twice) and that it produces an easily assessed measurement (not an interpretation) that was very reliable between observers (kappa 0.98–1.0, even with a non-expert observers).^16,17^ However, PC-MRI is a very recent addition, not yet widely available and has not yet been used to define CNO in any study. Also, PC-MRI seems to only be able to distinguish between CNO and conventional stenosis, not between other degrees of stenosis.^17^ See Supplemental Material for further details, including a discussion about why US and CTA measurement thresholds are not good enough for clinical use.

### Effect of CNO diagnostics on CNO epidemiology

Overall, the prevalence of CNO varied significantly between studies and was clearly affected by use of CNO diagnostics and to some degree when using selection criteria that can exclude CNOs to a varying degree (such as requiring treatment). In patient series with “good” CNO diagnostics, the prevalence findings indicated that CNO is common, occurring in 30% of cases with ⩾50% stenosis. Although many of these patient-series studies were from the same group, their findings aligned with the 27% (716/2667, Supplemental Table 5) reported in the 13 single-center CEA/CAS studies that had “good” diagnostics from other groups. The CNO prevalence in NASCET/ECST was lower than these real-world CEA/CAS series,^1^ but this might very well be a selection bias (well-known to exist in trials), especially as very few (6%) of CNOs had full collapse in these trials. Thus, CNOs are not “rare,” “relatively rare,” or “⩽10% of cases” (with varying definition) as described in 13 studies.^8,9,13,35–44^ In contrast, CNO has only been described as “common” (or similar) after a 2022 epidemiological assessment first described it as such,^45^ and then only twice by authors not involved in that assessment.^12,20^

### Effect of CNO diagnostics on CNO management

All current guidelines foremost recommend conservative treatment for symptomatic CNO, with some variations between them.^5–7^ This recommendation is very reasonable as all studies with “good” CNO diagnostics that compare revascularization with conservative management have found no benefit with CEA/CAS (Table 3). In contrast, four recent meta-analyses found that CEA/CAS should be considered for CNO.^8,9,12,35^ These did not take any aspects of diagnostics into account, and some had severe design issues, detailed in Supplemental Material. Further discussion is available in the Supplemental Material.

### Previous and likely current routine CNO diagnostics, and its effects on management

Reproducing NASCET/ECST diagnostics is reasonably the goal for routine diagnostics of carotid stenosis. This includes “good” CNO diagnostics. Thus, the assumption should be that use of “good” CNO diagnostics is widespread. How often “good” diagnostics is actually used in clinical routine is unknown, but there is ample evidence to suggest it is rarely used, as detailed in the Supplemental Material. Thus, CNOs seems to have been, and likely still are, systematically mistaken for conventional stenosis^19,20,22,25^ and therefore most likely be subjected to unnecessary CEA/CAS. This is our own experience when applying expert CTA assessments in retrospect.^27^ Indeed, in series with “good” CNO diagnostics, there is no relevant difference in CNO prevalence between unselected patient series (30%) and CEA/CAS series (27%), indicating systematic treatment of CNOs even when the diagnosis is known.

### Future studies and standards

**Figure 3.**
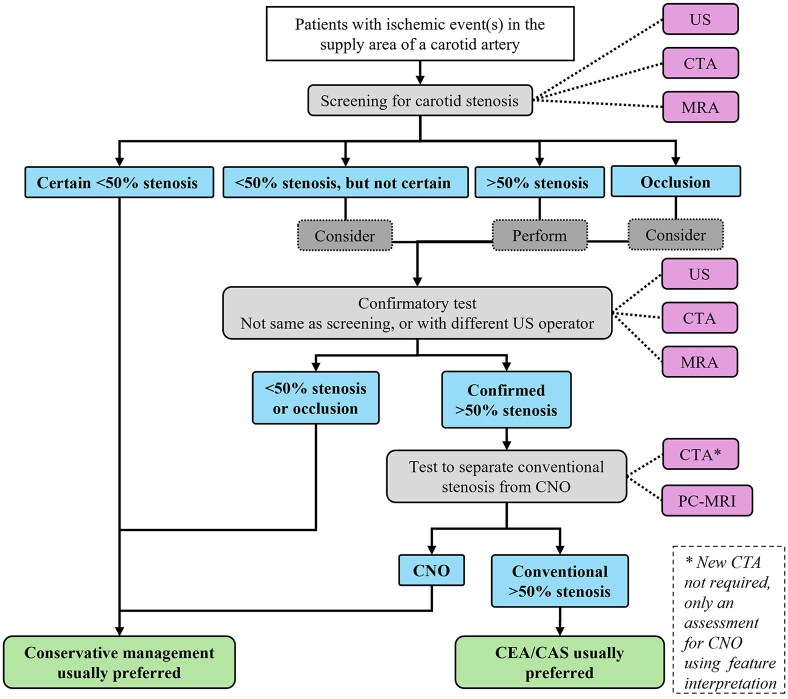
Suggested diagnostic pathway for carotid stenosis. The steps covered in this review are what happens after a >50% stenosis is confirmed and that one should consider a confirmatory test for occlusions. All other steps are taken from recent guidelines^7^ except to consider a confirmatory test when the diagnosis of <50% is uncertain, which is an opinion based on clinical reasoning (screening tests can be false negative). CNO: carotid near-occlusion; CTA: computed tomography angiography; MRA: magnetic resonance angiography (i.e. traditional lumen assessment, preferably contrast-enhanced); PC-MRI: phase-contrast magnetic resonance imaging; US: ultrasound.

Clinical validation of that conservative management of symptomatic CNO diagnosed with PC-MRI is warranted, this could be in the form of a registry or a randomized trial. The pros and cons of such a registry and the rationale for such a trial are detailed in the Supplemental Material. How to approach CNO diagnostics in current clinical routine is summarized in Figure 3 and also detailed in the Supplemental Material. After our systematic search, a study suggesting the use of either sensitive or specific US criteria to screen for the need for advanced CNO diagnostics (PC-MRI or CTA with feature interpretation) has been published.^46^ If validated, an improved algorithm will be possible in the future, where only some patients will require this advanced CNO diagnostics (Supplemental Figure 5). Thus, further studies into this type of feasible screening thresholds for US or CTA measurements are warranted.

Many of central studies for diagnostics and epidemiology were performed foremost by us and to some extent by group García-Pastor. This is problematic in two ways. First, it might cause confirmation bias in this review when interpreting the field. Second, there is a lack of external validation. Both the issues are reasonably solved by more groups performing CNO research (reviews and original studies).

A reporting standard is warranted: Report clearly if both CNO with and without full collapse is intended. Present group-level (mean or median) measurements of stenosis, distal ICA, ICA ratio and/or ECA ratio (or similar flow findings if using PC-MRI) to allow for comparisons between studies (discussed further in the Supplemental Material). In addition to clinical outcomes, presenting the long-term patency of the artery should be considered.

## Conclusions

No easily used, accurate and readily available diagnostic method to diagnose CNO exists. Symptomatic CNO is common but seems to be systematically mistaken for conventional stenosis, resulting in unnecessary CEA/CAS. A randomized trial or a registry of symptomatic CNO (managed conservatively or with CEA/CAS) is warranted, but to be relevant, it should use accurate and reproducible diagnostics (i.e. PC-MRI). Prognosis and management of asymptomatic CNO is virtually unstudied as most relevant studies have not separated CNOs from conventional stenosis in the analyses, so the relevance of CNO diagnostics is unclear for asymptomatic carotid stenosis.

## Supplementary Material

sj-pdf-1-eso_23969873251355158

## Data Availability

All data is publicly available in respective publication
